# Micro RNA 146a-5p expression in Kidney transplant recipients with delayed graft function

**DOI:** 10.1590/2175-8239-JBN-2018-0098

**Published:** 2018-11-08

**Authors:** Patricia Milhoransa, Carolina Caruccio Montanari, Rosangela Montenegro, Roberto Ceratti Manfro

**Affiliations:** 1 Universidade Federal do Rio Grande do Sul Programa de Pós-Graduação em Medicina: Ciências Médicas Porto AlegreRS Brasil Universidade Federal do Rio Grande do Sul, Programa de Pós-Graduação em Medicina: Ciências Médicas, Porto Alegre, RS, Brasil.; 2 Unidade de Transplante Renal Hospital de Clínicas de Porto Alegre Divisão de Nefrologia Porto AlegreRS Brasil Hospital de Clínicas de Porto Alegre, Unidade de Transplante Renal, Divisão de Nefrologia, Porto Alegre, RS, Brasil.

**Keywords:** Kidney transplantation, Graft Rejection, Delayed Graft Function, Blood, Biopsy, MicroRNAs, Biomarkers, Molecular Diagnostic Techniques

## Abstract

**Introduction:**

The development of novel non-invasive biomarkers of kidney graft dysfunction, especially in the course of the delayed graft function period would be an important step forward in the clinical practice of kidney transplantation.

**Methods:**

We evaluated by RT-PCR the expression of miRNA-146 to -5p ribonucleic micro-acids (miRNAs) in the peripheral blood and renal tissue obtained from kidney transplant recipients who underwent a surveillance graft biopsy during the period of delayed graft function.

**Results:**

In biopsy samples, the expression of miR-146a-5p was significantly increased in the group of patients with delayed graft function (DGF) (n = 33) versus stables patients (STA) (n = 13) and patients with acute rejection (AR) (n = 9) (*p* = 0.008). In peripheral blood samples, a non-significant increase of miR-146a-5p expression was found in the DGF group versus STA and AR groups (*p* = 0.083). No significant correlation was found between levels of expression in biopsy and plasma. ROC curve analysis revealed an AUC of 0.75 (95% CI: 0.62-0.88) for the renal tissue expression and 0.67 (95% CI 0.52-0.81) for the peripheral blood expression.

**Conclusion:**

We conclude that miR-146a-5p expression has a distinct pattern in the renal tissue and perhaps in the peripheral blood in the setting of DGF. Further refinements and strategies for studies should be developed in the field of non-invasive molecular diagnosis of kidney graft dysfunction.

## INTRODUCTION

Renal transplantation is the treatment of choice for many patients with end-stage renal impairment^1^^,^^2^. It offers a significant increase in life expectancy and quality of life of patients with end-stage renal function^3^. However, as evidenced since the beginning of organ transplants, tissues and organs of genetically distinct individuals lose their functions through a rejection process that is mediated by the immune system. Such process is only partially controlled by modifying the receptor immune response with immunosuppressive drugs and biological agents^3^^-^5.

In the last century, the mechanisms of alloimmune response were elucidated and immunosuppressive drugs capable of preventing rejection were developed turning organ transplantation into a clinical reality^6^^,^^7^.

In kidney transplantation, injury due to ischemia and reperfusion is an inevitable process resulting many times in delayed graft function (DGF) that is currently characterized by the need of dialysis within the first week after transplantation. Ischemia and reperfusion injury (IRI) also promotes activation of the innate and adaptive responses of the immune system, leading to processes with great potential to produce significant graft harm. It is believed that these injuries also facilitate mechanisms of acute rejection (AR) and act by programming gene, metabolic, and tissue changes that culminate in tissue graft fibrosis and chronic loss of function^8^^,^^9^.

AR, a frequent and ominous complication of organ transplantation, is still considered a risk factor for early and late graft loss^10^. Currently, the clinical phenotypes of rejection are well elucidated in clinical practice. Rejection is usually evidenced by the organ dysfunction, which leads to a graft biopsy that is classified by histological alterations^11^. Its evolution is difficult to predict and the histological findings observed in renal tissue obtained are still considered the best predictors^11^^,^^12^. Biopsy on the other hand is associated with a variety of complications. In addition, it is costly, has representativeness issues, and is subject to interpretation variability. Nevertheless, in current practice, it is still the gold standard for the diagnosis of renal graft dysfunctions^12^^,^^13^.

Graft damage classification is done through the *Banff* classification, a standard international consensus of nomenclatures and specific criteria for the histological characterization of organ rejection, in which T cell mediated rejection or antibody mediated rejection are diagnosed based on empirical rules and the lesions are graded semi-quantitatively^14^. Briefly, diagnoses according to this classification may be grouped as borderline rejection, acute, tubulointerstitial or vascular cell rejections of different severities, or as antibody-mediated acute rejection, characterized by histological findings, presence of donor antibodies anti-HLA and by the labeling of C4d in the peritubular capillaries^14^.

Accurate non-invasive biomarkers are an unmet need in the clinical practice of organ transplantation. Most of the work with molecular biomarkers has been done analyzing messenger RNA expression^15^^,^^16^. An important discovery of molecular biology in recent years is the micro-RNAs (miRNAs)^17^^,^^18^. They consist of small conserved and non-RNA coding fragments of approximately 25 nucleotides, which inhibit transcription of mRNA, are induced by translational depression or degradation of mRNA, and are responsible for regulating gene expression^18^^-^20.

Many microRNAs are involved in the development or progression of chronic or acute kidney disease in patients or animal models. hsa-miR-146a-5p was upregulated in patients with FSGS (focal segmental glomerulosclerosis) and MPGN (membranoproliferative GN) compared with patients with DN (diabetic nephropathy). miR-146a was modulated in an experimental model of renal I/R in mice and in patients with IgA nephropathy, where its levels in renal tissue and urine were correlated with injury severity. It is important to emphasize that miR-146a-5p, which demonstrated a very high diagnostic value in ICU (intensive care unit) patients, presents a strong and significant downregulation during early AKI (acute kidney injury). Thus, this miRNA could be considered a precise and early AKI diagnostic tool in several clinical contexts^18^^-^21.

Cell-free circulating miRNAs are present in various body fluids, such as serum, plasma, and urine, and in DN may reflect responses to various pathophysiological stresses. Urine is an ideal source of biomarkers for renal diseases and several studies have indicated miRNAs as potential biomarkers for diagnosis and monitoring of IgA nephropathy (IgAN). miRNAs are present in urine in a remarkably stable form, packaged in extracellular vesicles, predominantly exosomes. Urinary exosomes were successfully isolated to obtain exosomal miRNAs, and miR-146a may potentially serve as novel non-invasive biomarker for IgAN.

MicroRNA 146a-5p acts as a mediator of the renal tubular response to the ischemia-reperfusion injury, limiting the inflammatory process in this setting (21). In the present study, we quantitatively assessed miR-146a-5p in the renal tissues and peripheral blood lymphocytes of kidney transplant recipients with delayed graft function. We hypothesized that miR-146a-5p would present enhanced transcription signaling in recipients with DGF compared to patients with stable function and those with AR at two compartments, peripheral blood and renal graft tissue.

## METHODS

### PATIENTS

In order to obtain adequate statistical power, with an estimated AR incidence of 40% in patients with acute graft dysfunction and of 20% in patients without acute graft dysfunction, a proposed sample size of 55 patients that underwent renal graft biopsies was calculated and enrolled in the study. The sample size was calculated following the parameters: a) study power of 80%; b) Pα = 0.05; c) Pβ: 0.20; d) magnitude of the difference: 50%.

Peripheral blood was also obtained from these patients for miRNA analysis. Thirty-three patients had DGF when the biopsies were performed (surveillance biopsies), nine had acute graft dysfunction (indication biopsies) that was attributed to AR, and 13 were normal protocol biopsies obtained at three months after transplantation. The study was conducted at the Renal Transplantation Unit, Division of Nephrology, *Hospital de Clínicas de Porto Alegre*, Brazil, between May 2013 and April 2017. All patients provided written informed consent for their participation.

All patients were on immunosuppression consisting of a combination of corticosteroids, sodium mycophenolate, and calcineurin inhibitors. Either anti-IL2 receptor antibodies (Basiliximab^®^) or rabbit anti-thymocyte globulin (Thymoglobulin^®^) induction therapy was used in all deceased-donor graft recipients and for all living-donor graft recipients considered at increased risk of rejection.

The ethical and methodological aspects of this study were approved by the Hospital de Clínicas de Porto Alegre Research Ethics.

### SAMPLES

Biopsies were performed percutaneously, under real-time ultrasound guidance, using a semi-automatic biopsy gun with a 16G needle. At the time of biopsy, all patients had well-controlled blood pressure and all parameters of the coagulation panel (obtained no more than 24 hours before) within normal limits. Before renal biopsy, a comprehensive workup was performed to rule out obstructive or vascular issues, urinary fistula, infection, or drug toxicity as causes of graft dysfunction.

### SPECIMEN COLLECTION AND PREPARATION

Two renal cortex fragments were collected during each biopsy procedure. One-third of one of these fragments was placed in a microtube, flash-frozen by submerging in liquid nitrogen, and stored at -80°C. Peripheral blood samples (5 mL collected into EDTA-containing tubes) were obtained immediately before the biopsies.

For cell separation, both sample types were rinsed and processed to concentrate the cells of interest (blood). In the case of blood samples, 2-mL aliquots were transferred to sterile, 12-mL Falcon tubes to which 10 mL of erythrocyte-lysing Buffer EL (Qiagen Inc., Chatsworth, CA, USA) were added, followed by 21 minutes of incubation on ice with intermittent vortexing every 7 minutes. After this step, the samples were centrifuged at 1800 rpm for 10 minutes, leaving a pellet that contained the cells of interest at the bottom of the tube. The pellet was preserved and the supernatant discarded. The pellet was then resuspended in 1.5 mL of Buffer EL, transferred to microtubes, further centrifuged for 10 minutes at 10,000 rpm, the supernatant discarded, and the resulting cell concentrate frozen at -80°C.

### MICRO RNA PROCESSING

Micro RNAs were extracted from samples using the *mir*Vana™ PARIS™ commercial kit (Ambion^®^, Life Technologies Corporation). Briefly, cell concentrate/sediment was dissolved or fragmented with 500 µL of buffer in a dispersing machine (ULTRA-TURRAX T 10 basic - IKA, Campinas, SP, Brazil) and eluted in 60 µL of water for injection preheated to 95°C, in accordance with manufacturer instructions.

Samples were resuspended in 500 µL of ice-cold Cell Disruption Buffer and homogenized with a pipette. The lysate was transferred to 2-mL microtubes to which 500 µL of denaturing solution preheated to 37°C was added, homogenized again, and incubated in ice for 5 minutes. After incubation, 1 mL of the lower phase of the acid-phenol:chloroform provided in the kit was added, the lysate vigorously vortexed for 1 minute, and then centrifuged at 13,000 rpm for 5 minutes for organic phase separation. The phase of interest (the upper phase) was then collected, measured (maximum volume 600 µL), and transferred to a fresh microtube, to which was added 100% ethanol at room temperature corresponding to one-third of the obtained volume. The resulting solution was homogenized and 700 µL transferred to the filtering apparatus provided in the kit. This apparatus was then centrifuged at 10,000 rpm for 30 seconds. The resulting filtrate contained the desired miRNAs. Ethanol (466 µL) was added to this fluid, which was passed through a second filter, and the washing process begun. This process consisted of the addition of 700 µL of Wash Solution 1, centrifuging for 15 seconds at 10,000 rpm, addition of 500 µL of Wash Solution 2/3, and repeating the two preceding steps. After discarding the flow-through, the apparatus is centrifuged for 1 minute to remove any residual reagent, the column is transferred into a fresh collection tube, and the miRNAs are collected by eluting with 60 µL of water for injection heated to 95ºC. The eluate was then centrifuged at 10,000 rpm for 50 seconds and stored at -80ºC until the next stage.

The concentration of extracted miRNA was quantified in a full-spectrum spectrophotometer (220-750nm) with sample retention technology (Nanodrop 1000, Thermo Fischer Scientific, Wilmington, DE, USA). The nucleic acid concentration is expressed in ng/µL based on optical density at 260 nm, and purity is calculated based on the A260/280 and A260/230 ratios. A ratio of approximately 2.0 is generally accepted as "pure" RNA. Samples were considered viable if they had a concentration of at least 2 ng/µL. All samples with a higher concentration were diluted to this concentration in a 50 µL volume of nuclease-free water.

### AMPLIFICATION AND DETECTION

The following specific TaqMan primers (Applied Biosystems^®^) were used for real-time reverse transcription polymerase chain reaction (RT-PCR): miR146a-5p 4427975/000468; RNU-48 4427975/001006 and Cel-miR-39-5p 4427975/464312. The endogenous control used for sample normalization was synthetic exogenous control Cel-miR-39 from *C. elegans* (Qiagen, catalog number MSY0000010), which was spiked into samples before the reverse transcription stage in a 0.5 µL volume at a 50 pM concentration.

### COMPLEMENTARY DNA

The complementary DNA (cDNA) formation stage was carried out with the TaqMan MicroRNA RT kit (Applied Biosystems^®^) as per manufacturer instructions, using 8 µL of reaction mix: 0.12 µL of 100mM dNTPs, 0.8 µL of MultiScribe™ reverse transcriptase (50 U/µL), 1.2 µL of enzyme buffer, 0.144 µL of RNase inhibitor (20 U/µL), 3.40 µL of nuclease-free water, and 2.4 µL of target-specific primer, to which 4 µL of miRNA were added, finalizing a total volume of 12 µL. For synthesis, the samples were incubated at 16ºC for 30 minutes, at 42ºC for 30 minutes, and at 85ºC for 5 minutes, and then stored at -20ºC until the time for RT-PCR.

### REVERSE TRANSCRIPTION POLYMERASE CHAIN REACTION

RT-qPCR which consisted of the amplification of 2µL cDNA using 5.0µL of TaqMan Universal PCR Master Mix (Applied Biosystems^®^), 0.5 µL of specific primers, and 2.5 µL of nuclease-free water, in a final reaction volume of 10 µL. Analyses of miRNA expression were performed using TaqMan MicroRNA Assay and individual TaqMan MicroRNA Assays (TaqManMicroRNA Assay, Applied Biosystems, Foster City CA, USA) for miR-146a-5p a Step-One Real Time PCR 48-welloptical plate (Applied Biosystems, Foster City CA, USA)^22^.

One endogenous miRNAs (RNU48) was analyzed, and the expression biopsy data were normalized against RNU48.The data were presented as the relative quantity of target miRNA normalized to endogenous. The exogenous control used was a cel-miR-39 of blood samples.

The controls and normalizers were selected according to literature and with the help of the scientific advice of Thermo Fischer Scientific (https://www.thermofisher.com/br).

The cycle threshold (Ct) was calculated automatically using software. miRNAs expression was normalized using the 2^-∆∆Ct^ method described by Livak and Schmittgen^22^.

### STATISTICAL ANALYSES

Asymmetrically distributed variables are reported as medians and interquartile ranges, whereas symmetrically distributed variables are reported as means ± standard deviations. The Kruskal-Wallis and Mann-Whitney *U* tests were used for paired-samples analysis of variance and for between-group analysis. Spearman's correlation was used to see the association between two variables. Qualitative data are expressed as absolute and relative counts, and the chi-square or Fisher's exact tests were used for between-group analyses. All tests were two-tailed and a *p*-value < 0.05 was defined as statistically significant. All analyses were carried out in PASW Statistics 21.0 (SPSS Inc., Chicago, IL, USA).

## RESULTS

Diagnostic classification was achieved by a combination of the clinical assessment, response to specific therapy and biopsy findings, as interpreted per the Banff 2013 classification^14^. Fifty-five patients were divided between three groups according to the established diagnosis. Thirty-three patients were on DGF, nine were having an AR episode, and thirteen were patients with stable graft function that underwent a protocol biopsy. For all patients, a biopsy and a peripheral blood sample were obtained and processed for miRNA 146a-5p expression. Demographic data are shown in Table 1. Only serum creatinine levels (*p* = 0.022) and time elapsed from transplant to biopsy (*p* = 0.001) differed significantly between groups. Serum creatinine was significantly elevated in the DGF group and time to biopsy was longer in the DGF group compared to the stable group. As expected, time to biopsy was also longer in the stable group compared to the other two groups. The differences observed in the initial immunosuppression regimens did not reach statistical significance (*p* = 0.069).

**Table 1:** Demographic profile of studied patients and transplant variables

Variables	Stable n = 13	DGF n = 33	AR n = 9	*p*
Age (years; mean±SD)	48.3±12.2	46.8±14.5	42.8±11.6	0.632*
Sex (male/female)	9/4	15/18	4/5	0.318**
Race (white/nonwhite)	11/2	28/5	8/1	0.950**
Donor age (years; mean+SD)	45.6±10.4	46±16.9	42.8±17	0.864*
Donor sex (male/female)	4/9	17/16	4/5	0.444**
Early graft dysfunction (yes/group total)	9/4	29/4	7/2	0.434**
HLA mismatches (A, B, DR; mean+SD)	2.53±0.5	2.2±0.8	2.5±0.5	0.252*
Last PRA (%; median (IQR))				
Class I	1 (0-23.5)^a^	7.0 (0-21.5)^a^	11 (0-24.1)^a^	0.430*
Class II	2 (0-28.5)^a^	1.5 (0-22)^a^	2.5 (0-24)^a^	0.930*
Initial immunosuppression (n; %)				0.069**
No induction	6 (46.2)	10 (30.3)	4 (44.4)	0.518*
Rabbit anti-thymocyte globulin	1 (7.7.)	16 (50)	4 (44.4)	0.029*
Basiliximab	6 (46.2)	6 (18.8)	1 (11.1)	0.091*
Cold ischemia time (h; mean+ SD)	20+5.1	25.2+7.7	21.8+7.4	0.196*
Underlying renal disease (n; %)				0.509**
Unknown	6 (46.2)	7 (22.6)	4 (50)	0.165*
HTN	5 (38.5)	13 (39.4)	4 (44.4)	0.955*
DM	3 (23.1)	8 (24.2)	0 (0)	0.259*
HTN+DM	1 (8.3)	4 (12.5)	0 (0)	0.554*
APKD	2 (16.7)	4 (12.5)	1 (12.5)	0.934*
Other	0 (0)	8 (25.8)	2 (25)	0.147*
Serum Creatinine at Biopsy (mg/dL; mean ± SD)	2.5±1.9^a^	5.7±4^b^	3.8±3.3^ab^	0.022*
Time to biopsy (days; median [IQR])	99 [86-116]^b^	14 [12-26]^a^	58 [14-331]^ab^	0.001***

SD: standard deviation; HLA: human leukocyte antigen; HTN: hypertension; DM: diabetes mellitus; APKD: autosomal dominant polycystic kidney disease;

* Analysis of variance (ANOVA);

** Pearson's chi-square;

*** Kruskal-Wallis test;

a,b Equal letters do not differ by the Tukey's (ANOVA) or Dunn's (Kruskal-Wallis) tests at the 5% level of significance.

**Figure 1:**
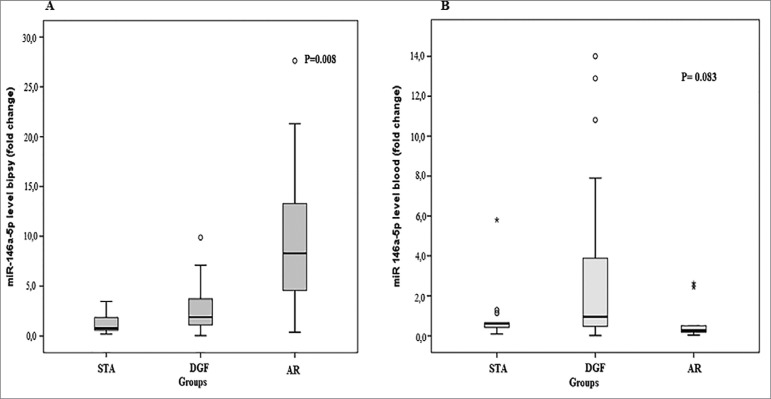
(A) Comparison of expression levels of miR-146a-5p in the renal tissue in the diagnostic categories (B) Comparison of the expression of the miR-146a-5p in peripheral blood in diagnostic categories.

In the renal tissue, microRNA 146a-5p was differentially expressed between groups. It was enhanced in the DGF group (median [IQR], 3.23 [1.46-5.74]) compared to the stable group (median [IQR], 0.78 [0.57-1.99]; *p* = 0.019). The differences observed in the comparisons between the DGF group (median [IQR], 3.23 [1.46-5.74]) and the AR group (median [IQR], 1.07 [0.43-2.11]; *p* = 0.106) and the comparison between the AR group and the stable group (median [IQR], 0.78 [0.57-1.99]; *p* = 1.0) were not statistically significant (Table 2 and Figure 1A).

**Table 2:** miR-146a-5p expression* in the renal tissue and peripheral blood in the groups of patients

	STABLE (n = 13)	DGF (n = 33)	AR (n = 9)	*p*-value
Biopsy miR-146a-5p	0.78 [0.57 - 1.99]	3.23 [1.46 - 5.74]	1.07 [0.43 - 2.11]	0.008
Peripheral Blood miR-146a-5p	0.62 [0.31 - 0.90]	0.96 [0.46 - 3.88]	0.27 [0.15 -1.47]	0.083

* Median [IQR]; P-values determined by Kruskal-Wallis/Mann-Whitney U test.

In the peripheral blood, the microRNA 146a-5p expression was heightened in the DGF group (median [IQR], 0.96 [0.46-3.88]) compared to AR group (median [IQR], 0.27 [0.15-1.47]) and the stable group (median [IQR], 0.62 [0.31-0.90]), however the differences were not statistically significant (*p* = 0.083) (Table 2 and Figure 1B).

**Figure 2:**
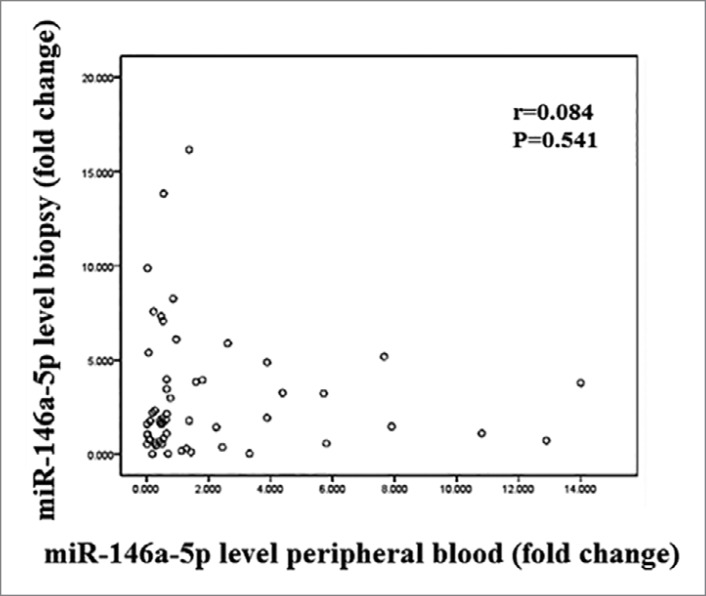
Correlation between expression of miR-146a-5p levels at the renal tissue and pheripheral blood.

As illustrated in Figure 2, no significant correlation was found between expression level of miR-146a-5p in different compartments, biopsy, and peripheral blood (r=0.084; *p* = 0.541).

**Figure 3:**
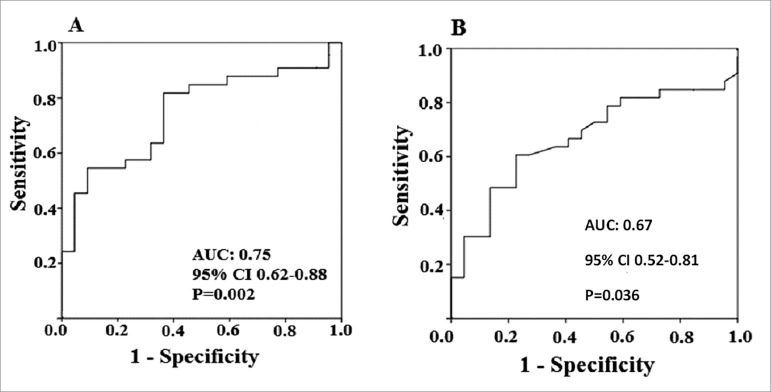
(A) ROC curve of the miR-146a-5p expression levels do the diagnoses of delayed graft function at the renal tissue; (B) ROC curve of the miR-146a-5p expression levels do the diagnoses of delayed graft function at the peripheral blood.

Receiver operating characteristic (ROC) curve of the biopsy analysis was plotted for assessment of diagnostic parameters of miRNA 146a-5p gene expression for the DGF diagnosis. The area under the curve (AUC) was 0.75 (95% CI: 0.62-0.88). Using a cutoff point of 1.64 at the ROC curve, i.e., a 64% increase in gene expression relative to controls, the obtained parameters were: sensitivity of 67.0%; specificity of 64.0%; positive predictive value of 73.3%; and negative predictive value of 56% (*p* = 0.002, Pearson's chi-square test) (Figure 3A). ROC curve of the peripheral blood analysis was plotted for assessment of diagnostic parameters of miRNA 146a-5p gene expression for the DGF diagnosis. The AUC for miR-146a-5p was 0.67 (95% CI 0.52-0.81). Using a cutoff point of 0.63 on the ROC curve, i.e., a 63% increase in gene expression relative to controls, the obtained parameters were: sensitivity of 64%; specificity of 64%; positive predictive value of 72.4%; and negative predictive value of 53.8% (*p* = 0.036, Pearson's chi-square test) (Figure 3B).

miRNAs have been used as biomarkers of pathophysiological processes such as the establishment of heart failure and neoplasms. The utility of miRNAs as biomarkers depends on several factors related to care during sample collection, processing, and storage. The conditions involved in the processing and storage of the miRNAs are of extreme importance for the integrity of these molecules be maintained until the moment of their analysis. Thus, the results obtained will not change due to methodological problems. Although the need for sample handling care is well known in the literature, it is still necessary to establish standard protocols for the collection, processing, and storage of this type of sample. In addition, the stability of the miRNAs during the storage period and in different conditions also does not have a consensus in the scientific community. This heterogeneity of procedures may be an important source of disagreement and questioning regarding the utility of miRNAs as disease biomarkers.

## DISCUSSION

Delayed graft function is a peculiar and very frequent form of acute kidney injury (AKI) that occurs immediately after renal transplantation. Its incidence is of around 25% in the United Network for Organ Sharing (UNOS) in North America but is substantially higher in Brazil^8^. Besides, Brazil carries a worse graft prognosis and perhaps patient survival. Similar to native kidney AKI survivors, DGF exhibits a significant risk of developing chronic kidney graft disease (CKD), with a course to accelerated end-stage renal disease^8^^,^^23^^-^26.

The ischemia and reperfusion injury (IRI) that occurs after renal transplantation is the main driver of the DGF clinical phenotype and perhaps, in its more serious forms, may lead to graft primary non-function. IRI also promotes activation of the innate and adaptive responses of the immune system, leading to processes that are potentially harmful to the kidney graft. It is believed that these injuries may also facilitate or trigger mechanisms of acute rejection (AR) and later, by gene reprogramming, metabolic and tissue changes may culminate in tissue graft fibrosis and chronic loss of function^8^^,^^9^.

Accurate and clinically useful biomarkers in AKI and IRI remain to be discovered. Early expression and accuracy are therefore critical parameters to be sought in AKI/IRI biomarker discovery^27^. Many disease states with significant inflammation, such as IRI and acute rejection have been associated with alterations in miR expression profiles^27^. MicroRNAs are key regulators of cell responses to many stimuli and can be secreted to the extracellular environment. Therefore, they can be detected in body fluids and this characteristic, among others, contribute to their great attention as disease biomarkers in many situations^27^. The search for noninvasive biomarkers reflecting intra-graft events opens important avenues for diagnosis, prognosis, and therapeutic monitoring in transplantation science. In the present study miR-146a-5p was assessed as potential biomarker of IRI that occurs in DGF after renal transplantation^21^^,^^27^.

The precursor pri-miR, miR-146a has been shown to be modulated in an experimental model of renal IRI in mice and in patients with IgA nephropathy. In this study, the miR levels in the renal tissue were found correlated with injury severity^21^. This precursor is processed into mature miR-146a-5p, which has been shown to be related to ischemia and renal IRI.

In present study, expression of miR-146a-5p in the renal tissue was significantly increased in biopsies of patients with DGF. In the sub-group analyses, the DGF group presented higher expression compared with patients with stable graft function. A non-significant difference was found in the comparison between the DGF and AR groups and very similar levels of expression were found in the comparison between the AR and stable groups. In the analysis of the miR-146a-5p obtained from the peripheral blood samples, the levels of expression were also higher in the DGF group, in comparison with the group of stable patients. In this analysis, the differences were of borderline statistical significance. Numerically, the DGF group presented higher levels of expression.

The miRNAs are involved in a variety of physiological and pathological processes, including stress response, inflammation, heart disease, neurodegenerative diseases (e.g. Alzheimer's and Parkinson's disease), autophagy, apoptosis, and various types of cancer. In the specific case of miRNAs exerting their regulatory role in cancer cells, these small RNA molecules can act as both oncogenes (activating the cell cycle) and tumor suppressor genes (inhibiting cell division), depending on the nature of the miRNA and the metabolic pathway in which they are involved. The association of characteristics such as biological function, presence in biological fluids, and stability places the miRNAs as promising biomarkers for the diagnosis and prognosis of various diseases.

Amrouche and colleagues found that this molecule might act as general regulator of the innate immune response in not only immune cells but also cells that are targeted by inflammation in human renal tissue and urine. They observed an increase in expression of this biomarker in patients with acute tubular necrosis early after transplantation compared to those who displayed normal allograft biopsy results^21^. Experimentally, they were able to demonstrate that renal ischemia induces tubular miR-146a expression in mouse kidneys after unilateral IRI and that, in comparison with the contralateral kidneys, the heightened levels of expression were still demonstrable up to 7 days after IRI. They also found that miR-146a was predominantly overexpressed in tubular cells. These results emphasize miR-146a as an important effector of the pathogenesis of the renal response to IRI injury 21.

Baker and collaborators studied the relative contributions of micro RNAs to the establishment of kidney disease. Analyzing human renal biopsies of patients with diabetic nephropathy, focal and segmental glomerulosclerosis, IGA nephropathy, membrane proliferative GN and controls they found that miR-146a-5p distinguished diabetic nephropathy from the other conditions and concluded that this molecule may be used as a biomarker of kidney diseases and is perhaps involved in disease mechanisms^28^. Fraile and collaborators analyzed miR-146a-5p expression levels in the sera of AKI and control individuals. They found that this microRNA is overexpressed in the serum of patients with AKI compared to healthy controls. It was also found that its expression levels in the renal tissue and urine are correlated with injury severity^29^^-^31.

Dziedic *et al*. investigated the correlation between plasma renalase concentration and miR-146a-5p expression in hemodialysis patients. Patients with simultaneous low miR-146a expression and high level of renalase were found to have a significantly longer survival time compared with other patients, and both miRNA-146a and renalase levels were estimated as independent prognostic factors of hemodialyzed patients' survival time^32^. Micro RNA 146 has been shown to be involved in the regulation and control of inflammatory processes. It is also involved in regulation of the immune system acting in a negative regulatory loop or in a feedback system that interferes in the inflammatory responses^33^^,^^34^. Renalase is secreted by many tissues including adipose tissue, cardiomyocytes, skeletal muscle, liver, central nervous system, and endothelium, besides its production in the kidney^35^. Overall, it is conceivable that miRNAs regulation of the renalase genes may participate in the physiopathogenesis of cardiovascular and metabolic diseases by altering its molecular basis^36^.

Tang *et al*. identified a miRNAs as regulator of target gene expression involved in shaping the immune response. These authors investigated the role of miR-146a in the pathogenesis of systemic lupus erythematosus and found that this microRNA, among others, act as a negative regulator of innate immunity in these patients. Further analysis showed that underexpression of miR-146a negatively correlates with clinical disease activity and with interferon (IFN) scores in patients with systemic lupus erythematosus. The microRNA miR-146a is a negative regulator of the IFN pathway; underexpression of miR-146a contributes to alterations in the type I IFN pathway in lupus patients by targeting key signaling proteins. They suggested that their findings provide potential novel strategies for therapeutic intervention^37^.

Previous research has shown that miRNAs can exit cells through exosomes and be transported in body fluids to other compartments where they may act as local regulators^38^^,^^39^. This biological property might, at least in part, explain the discrepancies between tissue and peripheral blood expression found in the present study. Alternatively, and perhaps more plausibly, the higher amounts found in tissue allows a better and more reliable detection of the microRNA. It is also possible that the evaluation in the urine would provide interesting results in terms of applicability of this molecule expression as a non-invasive biomarker. However, DGF recipients are frequently anuric ant that would be a relevant limitation for its application as a non-invasive biomarker in renal transplantation.

Among the weaknesses of the present study, perhaps the most important is the restricted number of individuals evaluated, which may have contributed to the negative results in the sub-group comparisons, more importantly in the peripheral blood analysis.

Although a miRNA panel capable of distinguishing among the various etiologies of dysfunction that can affect renal allografts has yet to be established, we identified miR-146a-5p as a potential biomarker for differentiating IRI, with the DGF clinical phenotype, from other conditions such as acute rejection. We suggest that the combined analysis of micro RNAs may lead to an accurate non-invasive diagnosis of kidney graft injuries.

In summary, further studies with other potential biomarkers of IRI and acute rejection, perhaps involving already surveyed microRNAs such as miR-146a-5p and miR-142-3 in different non-invasive samples might contribute to the development of accurate non-invasive biomarkers(s) for use in clinical organ transplantation^40^.
